# Older Adults’ Access to Pharmacological Treatment, a Human Right to Health: Scoping Review (2020–2025)

**DOI:** 10.3390/pharmacy14020046

**Published:** 2026-03-12

**Authors:** Doris Cardona, Valeria Santacruz-Restrepo, Juliana Madrigal-Cadavid, Alejandra Rendón-Montoya, Angela Segura-Cardona, Jorge Iván Estrada-Acevedo, Marcela Agudelo-Botero

**Affiliations:** 1 Independent Researcher, Medellín 050021, Colombia; 2 Value-Based Drug Safety Area, HelPharma, Medellín 050021, Colombia; valeria.santacruz@zentria.com.co; 3 I+D+i, Helpharma, Medellín 050021, Colombia; juliana.madrigal@zentria.com.co (J.M.-C.); alejandra.rendon@zentria.com.co (A.R.-M.); 4 Faculty of Medicine, CES University, Medellín 055420, Colombia; asegura@ces.edu.co; 5 Health Care Management Area, HelPharma, Medellín 050021, Colombia; jorge.estrada@zentria.com.co; 6 Policy, Population and Health Research Center, School of Medicine, National Autonomous University of Mexico, Mexico City 04510, Mexico

**Keywords:** right to health, older adults, health services accessibility, universal health services access, chronic disease, health services access barriers, aged

## Abstract

Background: Limitations in timely and equitable access to essential medicines among older adults not only constitute a clinical barrier to the effective management of chronic conditions, but also represent a violation of the fundamental right to life, health and the principles of dignity, equality and non-discrimination that safeguard this population within the framework of human rights. Objective: To examine access to essential medicines for older adults with high-cost chronic conditions as a constitutive dimension of the fundamental rights to health, life and human dignity, in accordance with international human rights standards. Design: A literature review was conducted of articles published between 2020 and March 2025 in five databases, using the search terms: “pharmacological treatment,” “access to health,” “chronic diseases,” and “barriers to access.” After evaluating the inclusion criteria (language and year) and exclusion criteria (case studies), 12 articles were selected. A narrative synthesis was performed on the following aspects: application of the principles of the right to health, barriers to access, and rights violated or at risk. Results: The expansion of health coverage faces several barriers that violate fundamental principles of the right to health: equity, accessibility to medical advances, and long-term, quality, and specialized services, thus limiting autonomy. In conclusion, guaranteeing access to pharmacological treatments in old age will contribute to building more just and humane societies through public policies on coverage and pharmaceutical education, the simplification of treatment regimens, and the implementation of programs that allow people to age with dignity, considering health a human right based on equality and non-discrimination, participation and transparency.

## 1. Introduction

The Universal Declaration of Human Rights advocates for the freedom, equality, and dignity of all people, without distinction of any kind [1], including older persons [2]. Health is an inclusive right that encompasses equitable access to health services, with availability, quality, and quantity, including essential medicines, without any discrimination based on age (ageism), sex (sexism), economic status (economic discrimination), ethnicity (racism), health status (ableism), or the combination of several (multiple discrimination) [3]. The World Health Organization has consistently affirmed that access to essential medicines is a core component of universal health coverage and a prerequisite for the effective realization of the right to health [4,5]. Consequently, economic, geographic, or administrative barriers restricting pharmacological access in older age should be understood not merely as health system deficiencies but as failures to meet States’ human rights obligations. A lack of medical care, and the denial of medications, treatments and diagnoses, in addition to negative attitudes by health professionals, violate their right to health [6,7,8,9].

Treatments for chronic disease care require a long time, which becomes a barrier to timely access to medications [10], and the diagnosis and detection of conditions [11,12], in older adults [13], increasing the cost of the health system [14] and out-of-pocket expenses [15,16]. Access to medicines includes: opportunity (arriving in time to have a positive impact on the evolution of the disease); quality and quantity; effectiveness and efficiency; cost (people can obtain it without affecting their living conditions); and the level of information (adequate and understandable for users) [6], but the World Health Organization (WHO) recognizes barriers to obtaining it: cost, low quality, inadequate use, acquisition problems and weaknesses in health systems, mainly in low-income countries [17], in addition to inequity in access [18], shortage of system resources, lack of qualified professionals, exclusion, stigma and discrimination, among others [19].

The high cost and shortage in the supply of essential medicines are barriers to accessing treatments [6,20], required in chronic diseases and geriatric syndromes [21]. Consequently, an increase in the number of older people who have more morbid states generates great challenges to guarantee healthy aging in society [22,23].

Population aging increases due to demographic transition (declining fertility, increased life expectancy) [24] and increasing access to health services for older patients due to multimorbidity, geriatric syndromes, frailty, cognitive decline, and/or pain [25,26,27,28]. Therefore, insufficient and effective access to treatment would lead to a lack of adherence [14] and violate human dignity [24].

From a public health perspective, the lack of access to essential medicines not only violates a fundamental right but also perpetuates structural inequalities and deepens the burden of disease in aging populations. Therefore, expressly recognizing the right of this population group to receive comprehensive health services that include the timely provision of medicines necessary for the treatment of chronic diseases and age-related conditions is crucial, given the limitations on timely, continuous, and equitable access to essential medicines. These are legal obligations derived from international human rights law [29,30,31], and this is the motivation for this review, which seeks to examine access to essential medicines for older adults with high-cost chronic diseases as a constitutive dimension of the fundamental rights to health, life and human dignity, in accordance with international human rights standards.

## 2. Materials and Methods

This scoping review is structured following the methodological recommendations proposed by Levac et al. (2010) [32] related to: identifying the research question (limitations in timely and equitable access to essential medicines among older adults constitute a barrier to the effective management of chronic conditions and are a violation of the fundamental rights to life, health, and the principles of dignity and equality), identifying and selecting relevant studies according to eligibility criteria (based on the PRISMA-ScR guidelines), and developing an analytical framework derived from the reviewed studies, which allows for the analysis of violated human rights, primarily the right to health.

**Protocol**. A literature review was conducted of articles published between 2020 and March 2025, following the PRISMA-ScR guidelines (Preferred Reporting Items for Systematic reviews and Meta-Analyses extension for Scoping Reviews) [33]. The completed checklist is provided as Table S1.

**Eligibility criteria.** We are looking for publications related to barriers in pharmacological access for the treatment of chronic diseases, particularly those of high cost to the health system suffered by older people (60 years and older), human rights, the right to health and barriers to access to medicines. The studies include different methodological approaches (8 quantitative, 3 qualitative, and 1 regulatory and analytical review), and different geographical contexts (3 Europe, 3 Asia, 5 America, and 1 Oceania).

**Information sources.** Five databases were searched: PubMed, Scopus, Web of Science, ScienceDirect, and Wiley Library. Search terms with Boolean connectors were used: “acceso a medicamentos” OR “access to medication”, “personas mayores” OR “older adults”, “enfermedades crónicas” OR “chronic diseases”, “alto costo” OR “high-cost”, “barreras” OR “barriers”, and “derecho a la salud” OR “right to health”.

**Figure 1:**
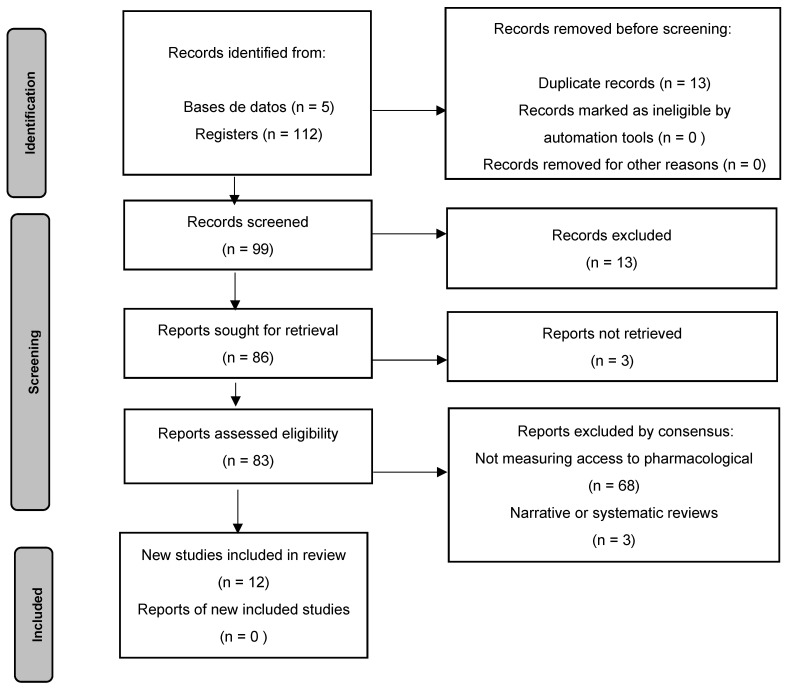
Flowchart of the process of identification, screening, eligibility, and inclusion of studies according to the PRISMA-2020 guideline.

**Search**. For the study selection process, the Rayyan software was used, an Artificial intelligence platform to which 112 relevant references were exported and, after removing 13 duplicates, 99 articles remained. Thirteen studies were excluded because they were not related to access to medicines. An attempt was made to retrieve full texts for the remaining 86 studies; 3 articles could not be retrieved, so they were excluded, and 83 abstracts were reviewed. Of these, 68 were excluded for not being related to the elderly population or not analyzing access to pharmacological treatment (addressing clinical adherence, effectiveness of treatments, etc.), leaving 12 articles included for convergent mixed methods. English and Spanish were included as inclusion criteria. Case reports and three (3) narrative or systematic reviews were excluded. Figure 1.

**Data items**. A data extraction matrix was designed that included: authors, type of study/methodological design, country/context, population/sample, right violated/at risk, main finding related to access to health, and human rights principles or approaches mentioned in each study (e.g., equitable access to health, quality services, right to healthcare, and telemedicine, among others).

**Data registration process.** An initial exploratory review was conducted by the first author between March and April, which helped to integrate different types of evidence without categorizing them, while maintaining the transparency and reproducibility of the study search and selection process. This identified 112 articles, and after removing 13 duplicates and 13 excluded articles, an attempt was made to retrieve the full texts of the remaining 86 studies. However, 3 studies could not be accessed and were therefore also excluded. Two reviewers then read 83 abstracts. When discrepancies arose between them, a third reviewer resolved them. Once the final 12 articles were identified, the extracted information was recorded in Table 1.

**Critical appraisal of individual sources of evidence.** This review consulted studies that demonstrated internal consistency in terms of selection, comparability, and results, using guidelines and checklists such as STROBE (Strengthening the Reporting of Observational Studies in Epidemiology) [34] and COREQ (Consolidated Criteria for Reporting Qualitative Research) [35].

**Synthesis of results**. With the 12 selected studies [36,37,38,39,40,41,42,43,44,45,46,47], a narrative synthesis of mixed methods was made in a convergent way where the data are combined and integrated simultaneously, in a descriptive way in the following aspects: (1) application of the principles of the right to health proposed by the United Nations: equality and non-discrimination, transparency, participation and responsibility [29], (2) barriers to access to pharmacological treatments, and (3) rights violated or at risk and an analysis according to ethical principles of justice towards the elderly population.

**Ethical considerations**. It is noted that the literature review was not presented to any ethics committee, since older adults were not surveyed, and the moral rights of the authors of the research were respected.

## 3. Results

### 3.1. Included Studies

The variety in the methodological approaches and contexts of the articles included allowed us to show an interest in addressing the problem of access to pharmacological treatments for the elderly. A direct parallel was made between the increase in population aging and chronic diseases, many of which are chronic conditions that demand more public and private resources for comprehensive care, as recommended by the universal values of “leaving no one behind” (United Nations and World Health Organization) to guarantee equity in health and the enjoyment of rights. 

Likewise, the studies included were carried out in different geographical contexts, showing that access to medicines is a generalized challenge for societies, mainly due to administrative difficulties in providing health services of quality and quantity. These are related to the multimorbidity of the elderly and the high costs of medications for chronic disease care, motivating an increase in research and strategies to reduce gaps in vulnerable populations over time, with more studies from 2024. The countries of origin were: Latin America (Chile), North America (United States), Asia (China, India and Taiwan), and Oceania (Australia).

Several studies analyzed general access to medications for the most prevalent chronic conditions, such as hypertension, diabetes, and cardiovascular disease. Others addressed specific events such as oral health and more global analyses on essential drugs for the treatment of noncommunicable diseases in old age, which present difficulties due to their distribution and equitable provision. Another aspect that is addressed in most of the articles is the explicit consideration of the right to health, mainly in the principles of equality and non-discrimination. They address equitable access to health and to preventive and curative treatments, without neglecting participation (autonomy, dignity, and continuity of care), and responsibility (universality and health coverage); however, the latter two are in smaller quantities [36,37,38,39,40,41,42,43,44,45,46,47]. Table 1.

**Table 1:** Selected articles and principles addressed.

Authors	Year	Country	Type of Study/Methodological Design	Population	Right Violated/at Risk	Principle Addressed	Main Findings
Li et al. [36]	2020	China	Observational (quantitative, cross-sectional)	5166	Timely hospitalization	Equality and non-discrimination	Multimorbidity and loss of functional capacity are barriers to accessing medical care, which is why they recommend home healthcare
Ronca et al. [37]	2020	Switzerland	Observational (quantitative, cross-sectional)	492	Access to specialized services	Transparency	Residents in rural and remote areas exhibit more geographic barriers to accessing specialized medical care
Taylor et al. [38]	2021	United States	Observational (quantitative, cross-sectional)	21,040	Access to oral health	Equality and non-discrimination—Transparency	Low income and lack of insurance are barriers to accessing dental care
Zurynski et al. [39]	2021	Australia	Observational (quantitative, cross-sectional)	1024	Affordability of healthcare services	Equality and non-discrimination—Transparency	Economic hardship is a barrier to accessing healthcare, medicines, and specialists
Melchiorre et al. [40]	2022	Italy	Qualitative (interviews)	120	Accessible primary care	Equality and non-discrimination—Transparency	The chronicity of diseases, public health services, low income, territorial distances and asymmetrical communication with doctors are barriers to healthcare
Tessier et al. [41]	2022	Switzerland	Regulatory and analytical review	N/A	Right to long-term care	Equality and Non-Discrimination—Responsibility	Proposes a system of solidarity and universal coverage throughout life, with public financing to prevent diseases and guarantee long-term care in old age, dependent on eliminating financial barriers, from a human rights perspective
Wardlow et al. [42]	2023	United States	Qualitative (Delphi technique)	40 experts	Senior-centered telemedicine	Participation	Develop guidelines to adapt telemedicine to the physical, cognitive, and social needs of older patients.
Badawoud et al. [43]	2024	United States	Observational (quantitative, cross-sectional)	107	Autonomy in health management	Participation	Inadequate knowledge of medications, the consumption of several of them simultaneously, and the lack of health education are barriers to the treatment of their diseases
Chen et al. [44]	2024	Taiwan	Observational (quantitative with clinical trial emulation)	4,972,228	Diagnosis and follow-up in mental health	Equality and Non-Discrimination—Responsibility	The rate of treatment is low in patients with depression and must be accompanied by adequate follow-up in vulnerable populations.
Smith et al. [45]	2024	United States	Observational (quantitative, cross-sectional)	1101 households	Access to telehealth	Equality and Non-Discrimination—Participation	The digital divide prevents the elderly, the chronically ill, Hispanics, and those living in rural areas without internet access from being barriers to accessing equitable healthcare.
Cabieses et al. [46]	2025	Chile	Qualitative (interviews)	29	Access to health technologies	Participation	Access to insulin pumps is limited by stigma, staff bias, costs, and geographic barriers
Halder & Kasemi [47]	2025	India	Observational (quantitative, cross-sectional)	400	Equitable access to healthcare	Equality and non-discrimination—Transparency	Older people prefer private services due to distrust or inefficiency of the public system, influenced by economic and social conditions

### 3.2. Principles of the Right to Health

The reviewed articles seek to apply the principles of the right to health proposed by the United Nations: equality and non-discrimination, transparency, participation, and accountability [29], as follows:

#### 3.2.1. Equality and Non-Discrimination 

This principle states that all people are equal before the law, must receive equal treatment, and must be treated in a respectful and dignified manner, without being discriminated against (or mistreated) according to specific conditions such as age, sex, illness, etc. In this sense, nine studies included aspects related to this principle; for example, Tessier [41] proposed universal coverage of the health system, care, and access to social protection throughout life as part of the human rights of older people, generating well-being and combating ageism (age discrimination). This universal, public and solidarity-based system contributes to healthy aging and accounts for the Sustainable Development Goals. Likewise, there are individual conditions of the elderly that lead them to not have fair access, such as the loss of functional capacity, multimorbidity [36], low income [38,39,40], mental illness [44], ethnicity [45] and residence in rural areas [37,45].

#### 3.2.2. Transparency

Transparency refers to the right to have information, and to have access to quality public services and in sufficient quantities. This right is not specifically addressed in the articles, but six of them mention different elements that were mainly related to access to information, such as geographical barriers [37]. Living in rural areas or with a lack of transportation [40] limits the enjoyment of affordable and easily accessible, basic and specialized healthcare [37,39]. In addition, older people who have a low or reduced level of education or are illiterate cannot access public information [40].

#### 3.2.3. Participation

This principle seeks to guarantee the right to make decisions that affect their lives, as is the case with the consumption of medications to prevent or treat the different chronic diseases suffered by the elderly, inviting patients to be an active part in the treatment of their ailments. The use of digital technology primarily for telehealth and/or telemedicine [42,45] requires that older adults take an active part and get involved, since it is not possible to do so in the case of access to pharmacological and specialized treatments. Special mention should be made of the study by Badawoud et al. [43] on the autonomy of the elderly to manage their diseases, due to inadequate knowledge of their medications, the simultaneous consumption of several of them, and the lack of health education.

#### 3.2.4. Responsibility

It is the principle that we must ensure that citizens’ rights are respected and protected, with accountability. None of the studies mentioned this principle explicitly, but three of them advocated the need for a supportive and universal health system (“leaving no one behind”), financed with public resources, allowing the prevention of diseases and healthcare access at all ages without any discrimination against the elderly. To guarantee human rights [41], continuity in the treatment of chronic diseases, which can be affected by the patient (individual, social, psychological, economic and health status), follow-up in the care and attention of vulnerable populations is needed [44].

### 3.3. Barriers to Access to Pharmacological Treatment

In summary, access to medications has several identified and some interrelated barriers in the 12 studies. Mainly, financial and economic barriers exist for vulnerable and low-income older adults to acquire pharmacological treatments. That is why several researchers recommend public financing of comprehensive, universal, and supportive health services that provide essential medicines and care for patients throughout life. Therefore, the availability and affordability of medicines for the care of high-cost diseases are compromised in countries with middle income and resources, and in those where the state cannot sustain the public health system.

Individual barriers also affect access to medicines and are related to the lack of knowledge or information about their diseases and treatments [43], and lack of knowledge of their rights and duties to access free or lower-cost medicines, given age, for example. That is why health information and communication are explicit in the right to health, through clear and precise guidance by health professionals, concerning complex therapeutic regimens and polypharmacy, which guarantees the improvement of their health. Unfortunately, many older people are subjected to discriminatory treatment, psychological violence, or multiple discrimination due to their age, physical health and mental health, among others.

There are also geographical barriers [37] that limit physical accessibility to medications, primarily for the elderly with reduced mobility or disability. The distance to health institutions [40]; the small number of pharmacies nearby; the absence of home delivery programs; few mobile dispensing units; long distances for residents in rural areas and dispersed territories; the lack of public transport and access roads that guarantee safe travel; and the concentration of specialists in large urban areas and main cities are some of the geographical barriers that limit access to pharmacological treatments [36].

Administrative and health system barriers are related to the numerous and complex institutional procedures and policies that make it unclear to access medication and care [41]; for example, requirements for authorizations of high-cost treatments, delays in the delivery of one or more drugs, fragmentation in the delivery of specialized services or complex treatments, assignment of multiple drugs for long-term chronic diseases, unclear adverse events, or drug interactions. Among other barriers, these add to the geographical and economic [38,39] barriers for the movement of older adults; they increase inequality and reduce their quality of life and well-being, and therefore violate their right to life and health.

### 3.4. Rights Violated or at Risk

The barriers identified imply the possible violation of several fundamental human rights, mainly the elderly’s right to health, which is compromised when they cannot access prescribed medication that is required to preserve their health and life. The right to health is the enjoyment of the highest level of health that can be achieved. Given their individual conditions, life history, culture, food, genetic load, context, and the availability of services, among other conditions, the lack of access to pharmacological treatments puts the right to life at risk. This violates the principle of “responsibility” by failing to protect the lives and integrity of elderly citizens, who are falling behind in their aspiration to enjoy a long life and healthy aging.

The studies reveal possible instances of discrimination and inequality in healthcare; for example, when the allocation of high-cost medications and treatments to younger patients is prioritized, citing a longer life expectancy and future contributions to society; when fewer resources are designated for the payment of professionals and geriatric institutions or adaptations for the physical accessibility of elderly people with reduced mobility; and when access to specialized services or telemedicine programs is limited because of language that is not very sympathetic to the elderly, or when the information provided is asymmetrical. These are ways of evidencing the violation of the right to adequate healthcare, as they manifest ageism or discrimination in healthcare institutions. It is a form of mistreatment and violence against the elderly person, based on age or their state of health, in the health field.

The right to social security is also violated and compromises their well-being and economic security. Several older adults must allocate personal and family financial resources, from their income and pensions, to obtain their medications and treatments with out-of-pocket expenses. This limits their basic daily sustenance and autonomy, violating their dignity by forcing them to renounce other priority expenses like housing, food, rest, and leisure, putting the right to enjoy an old age with a good quality of life at risk. This right is violated when healthcare is not covered with public or private resources, and the elderly must allocate their personal and family assets, by acquiring debts to sustain their treatments—mainly high-cost ones. This ultimately undermines their autonomy, their economic security, and their well-being. Because of this, some authors advocate for a public and universal health system that guarantees the care and follow-up of patients with chronic, multimorbid, and polymedicated diseases throughout their lives.

Finally, the barriers to access to high-cost drugs to treat chronic diseases are not only personal or administrative problems, as commonly shown. They also involve potential violations of the fundamental human rights of older adults: the right to health, life, equality, and social security, which together compromise human dignity, as they underlie all the rights mentioned in this review. Everyone deserves dignified treatment and access to healthcare throughout their life, which implies having access to pharmacological treatments, care, and respect for their health conditions. This means not feeling forgotten or like an economic burden on society and families. In other words, every barrier or obstacle to access specialized health services, timely hospitalization, early diagnosis, more affordable prescribed medications, and new health technologies threaten the dignity of the elderly.

## 4. Discussion

This review sought to identify access to pharmacological treatment for older adults with high-cost chronic diseases, highlighting barriers (various and of different nature) to access medicines for their ailments. It draws attention to the violation of human rights, with emphasis on the right to health, life, equality, and dignity.

The evidence found shows that, in practice, equitable access to health means accessibility to timely and comprehensive medical care, affordable health services, and the availability of medicines in quality and quantity. This presents different geographical, administrative, economic, financial, and personal barriers, which limit the application of the principles of the right to health, such as the following: equality and non-discrimination, with equal, respectful, fair, and barrier-free treatment; transparency in accessibility and affordability of information and services; individual and collective participation and commitment in the maintenance of health; and responsibility, which has generated tensions between reality and legality, with ethical implications that will be discussed.

A recurring concept in this review was access to healthcare. Some authors pointed out that the right to health implies providing quality care [36,41,47] and essential medications in the prescribed quantity [48]. Many seniors have low income and live in areas that are far from healthcare facilities [37,45]. Protecting this right implies taking legal and administrative measures to ensure the availability, accessibility and affordability of medicines, as indicated by international human rights guidelines and as stated verbatim in Sustainable Development Goal (SDG 3.8), “Achieve universal health coverage, including financial risk protection, access to quality essential health-care services and access to safe, effective, quality and affordable essential medicines and vaccines for all” [49].

However, many older adults see their rights violated due to economic barriers in acquiring high-cost medications without affecting collective rights [50], including the following: Administrative barriers—not having the quality and quantity of health services, both in public [51] and private sectors (many older adults turn to the private sector), to provide the needed care and medications [52]. Discriminatory practices by health professionals [53] and of the system itself, for fair and universal access for other groups, consistent with some of the articles reviewed [39]. Ignoring the universal values of sustainable development and human rights of the United Nations’ “Leave no one behind”, which seeks to eliminate forms of discrimination and inequality (multiple and interrelated) that diminish their ability to act as rights-holders [54].

This review does not address social justice directly, but it is implicit in equitable access to medications for the management of high-cost chronic diseases that affect a vulnerable group, such as the elderly, for whom this can make a life-or-death difference. To talk about health equity is to prioritize those who need it most according to the severity and progression of their diseases, and in turn, those who have the least capacity to stay healthy. Older adults see their rights violated due to their age and their state of health (multimorbidity and polypharmacy), which leads to them being discriminated against by health systems, professionals, society, and the State. Therefore, it is necessary, ethically speaking, to allocate sufficient resources to ensure their pharmacological therapies, more so if they are expensive, as is the case with long-term treatments of chronic diseases [55]. Any omission, reason, or justification for failing to provide adequate and timely treatment is neglect or abuse [56] and constitutes a social injustice.

Distributive justice is also included, given that resources are finite, so collective benefits must take precedence over individual rights, and high-cost pharmacological treatments for the care of chronic diseases imply the allocation of a high budget. Worldwide, there is an accelerated population aging, an increase in the prevalence of chronic diseases, and longer life expectancy, with international requirements to guarantee healthy aging [57,58]. So, how can we guarantee a fair distribution of health resources if about half of health spending should be invested in older adults? [59]. How can the right to health of the elderly be respected, without undermining the rights of other population groups that are the majority?

According to the principles of the right to health—transparency, participation, and responsibility—a fair distribution of resources could be made if health technologies that are easily accessible to all electronic devices (telehealth) are implemented, as proposed by several of the studies included in this review [42,45]. The inclusion of remote pharmaceutical care services is also an option [60]. It is therefore concluded that investment in health must also guarantee the benefit of the elderly, but based on clinical criteria and not based on age. Having state benefit plans [41], which invoke their responsibility to protect vulnerable groups (such as older adults) under the principles of distributive justice, thus avoids health disparities caused by economic and structural barriers to access treatment and diagnosis [61].

The review also shows that strengthening primary care, as mentioned by Melchiorre et al. [40], can mitigate geographical [37] and communication barriers [40]. Mentioned by several studies included in this review, bringing medication closer to older adults through home deliveries, community volunteers, and mobile units in rural areas, or encouraging telemedicine [42,45] and training health professionals, could reduce the barriers present in access to pharmacological treatments—advocating equality and non-discrimination, mentioned as the first principle of the right to health. However, many professionals face ethical dilemmas in deciding whether to prescribe a high-cost drug to older adults who do not have the resources to afford it or do not have access to public health services [62,63].

The findings of this review make a call to strengthen equity, justice, and inclusion in public policies towards old age, with a comprehensive approach. Given the multifactorial nature of the barriers evidenced in this review and the accelerated population aging and increase in older people with multiple and high-cost chronic diseases, more people will see their right to health violated. Therefore, eliminating barriers in access to pharmacological treatments is not only ethical, but also cost-effective in the future, especially if age is prevented from being used as an exclusive criterion for rationing expensive drugs, since that would infringe fundamental rights for reasons of age.

The authors also recognize the updated review as a strength. The following limitations were also noted in the studies on older adults: there was a lack of studies on the violation of human rights in health, little interest in addressing access to pharmacological treatments due to polypharmacy, limited data from low- and middle-income countries, design heterogeneity in the largest number of studies on access to health services, and a clear lack of studies quantifying barriers to access pharmacological treatment and their long-term impact, as well as demonstrating the cost of inaction (the cost of not providing medications to treat chronic diseases), such as complications, health effects, hospitalizations, increased consultations, and deaths.

## 5. Conclusions

The 12 studies included identified barriers to treatment access, including financial, economic, individual, geographic, and administrative barriers that prevent older adults from obtaining the medications they need to live a healthy and dignified life. These barriers are interrelated and create health inequities, primarily affecting the poorest, those living in remote areas, those with lower educational levels, those with multiple morbidities, and those most vulnerable to social discrimination, deepening health gaps in old age.

Guaranteeing access to pharmacological treatments in old age will contribute to building fairer and more humane societies, where aging does not mean losing rights, but enjoying the highest possible health. This is achieved through the strengthening of universal pharmaceutical coverage policies for essential and high-cost medicines, with caps on out-of-pocket spending, bringing dispensing closer to their homes, and avoiding shortages. It is also important to simplify procedures, guarantee the continuity of treatments, and train and raise awareness among health professionals. The implementation of strategies for telephone follow-up or home visits to the elderly with polypharmacy can be used to anticipate adverse events or a lack of adherence to treatments, and formulate research with a human rights perspective—emphasizing the right to health in the elderly, with equality and non-discrimination, participation, transparency, and responsibility.

## Data Availability

No new data were created or analyzed in this study.

## Supplementary Materials

The following supporting information can be downloaded at: https://www.mdpi.com/article/10.3390/pharmacy14020046/s1, Table S1: Preferred Reporting Items for Systematic reviews and Meta-Analyses extension for Scoping Reviews (PRISMA-ScR) Checklist

## Abbreviations

WHO	World Health Organization (WHO)
PRISMA	Preferred Reporting Items for Systematic Reviews and Meta-Analyses
SDG	Sustainable Development Goal
